# Editorial: Intellisense, guidance, control, and risk assessment of autonomous marine vehicles

**DOI:** 10.3389/fnbot.2023.1231853

**Published:** 2023-06-23

**Authors:** Guibing Zhu, Namkyun Im, Qiang Zhang

**Affiliations:** ^1^School of Naval Architecture and Maritime, Zhejiang Ocean University, Zhoushan, China; ^2^Department of Navigation Science, Mokpo National Maritime University, Mokpo, Republic of Korea; ^3^School of Navigation and Shipping, Shandong Jiaotong University, Jinan, China

**Keywords:** autonomous marine vehicles, intellisense technique, intelligent control technique, advance guidance method, navigation management

In recent years, with the rapid development of artificial intelligence, the internet of things, big data, and other high-tech technologies, along with their combination with marine vehicle and marine engineering, the development and research of intelligent/unmanned marine vehicles have received great attention from major shipbuilding and shipping countries throughout the world.

Autonomous marine vehicles are becoming an important aspect of future maritime transportation, and will eventually operate in the environment with an unmanned mode. In this context, the implementation of autonomous vehicle manipulation and control with safe, economical, and efficient navigation will become a prerequisite for current applications. In practice, its ultimate goal is to implement the various navigational tasks in the complex and changeable marine environment for a long time, safely, reliably, and independently. To do this, there are many problems involved in the autonomous navigation of autonomous marine vehicles, such as environmental perception, guidance, control, and decision-making, that need to be addressed. The focus of this Research Topic is on studies exploring the methods and paths using information technique, intelligent algorithms, etc., for realizing the autonomous and safe navigation capabilities of autonomous marine vehicles and improving their control performance. The research discusses the intellisense method, guidance techniques, advanced control techniques, and navigation risk management techniques of autonomous surface vehicles.

After a stringent peer review process, there were eight papers finally included in this Research Topic, which cover the following aspects: (1) Intellisense techniques and methods for autonomous surface vehicles, (2) Intelligent control methods for autonomous surface vehicles, and (3) Advanced guidance method and its application in the field of autonomous surface vehicles.

1. Intellisense techniques and methods for autonomous surface vehicles
  The article by Zhang et al. proposes a method based on the object detection model for recognizing vessel plate numbers in complicated sea environments applied to unmanned surface vessels (USVs) and establishes a USV platform including a number dataset in the South China Sea to evaluate the proposed method in an experiment. In the study by Bai et al., a lightweight deep learning detection model based on YOLOv5s is presented to resolve the issue on the emergency rescue of maritime accidents based on unmanned Aerial Vehicles.
2. Intelligent control methods for autonomous surface vehicles
  The article by Niu et al. presents a distributed constant bearing guidance and model-free disturbance rejection control method for formation tracking of autonomous surface vehicles subject to a fully unknown kinetic model. An application to formation control of autonomous surface vehicles is given to show the efficacy of the proposed integrated distributed constant bearing guidance and model-free disturbance rejection control. The article by Zhao et al. employs the line-of-sight (LOS) -based guidance method to resolve the control design issue of underactuated marine surface vessels, and then an online recorded data-based composite neural finite-time control scheme is proposed. The article by Guo et al. proposes an event-triggered fault-tolerant control method (PEFC) based on proportional logarithmic projection analysis. The simulation results showed that the method adopted can reduce the power output by 28.95% and the update frequency of power output by 75% compared with the traditional adaptive overdrive fault-tolerant control method. In Chen and Liu, an adaptive extended kalman filter algorithm based on innovation sequence is proposed to handle the influence on the observation accuracy caused by the gross error interference in estimating the speed and rotor position of a ship propulsion permanent magnet synchronous motor. Finally, the proposed algorithm is verified through simulation and experiment.
3. Advanced guidance method and its application in the field of autonomous surface vehicles
  The article by Zhuang and Chen proposes a ship route autonomous generation scheme for autonomous surface vehicles by AIS ship trajectory big data and improved multi-task long short-term memory artificial neural network. To solve the path planning of USVs, authors Gong et al. propose an improved differential evolution particle swarm optimization algorithm. Numerical simulation results show the proposed algorithm can effectively reduce the path intersection points, and thus greatly shorten the overall path length.

## Author contributions

GZ: conceptualization, methodology, formal analysis, and writing—original draft. NI and QZ: conceptualization, formal analysis, and writing—review and editing. All authors contributed to the article and approved the submitted version.

